# Silver Nanoparticles Combined With Naphthoquinones as an Effective Synergistic Strategy Against *Staphylococcus aureus*

**DOI:** 10.3389/fphar.2018.00816

**Published:** 2018-07-26

**Authors:** Marta Krychowiak, Anna Kawiak, Magdalena Narajczyk, Agnieszka Borowik, Aleksandra Królicka

**Affiliations:** ^1^Laboratory of Biologically Active Compounds, Intercollegiate Faculty of Biotechnology, Medical University of Gdańsk, University of Gdańsk, Gdańsk, Poland; ^2^Laboratory of Plant Protection and Biotechnology, Intercollegiate Faculty of Biotechnology UG and MUG, University of Gdańsk, Gdańsk, Poland; ^3^Laboratory of Electron Microscopy, Faculty of Biology, University of Gdańsk, Gdańsk, Poland; ^4^Laboratory of Biophysics, Intercollegiate Faculty of Biotechnology UG and MUG, University of Gdańsk, Gdańsk, Poland

**Keywords:** combination of antimicrobials, Droseraceae, plant secondary metabolite, 1, 4-naphthoquinone, antibiotic resistance, membrane damage, silver ions

## Abstract

*Staphylococcus aureus* is a human pathogen responsible for many antibiotic-resistant infections, for instance burn wound infections, which pose a threat to human life. Exploring possible synergy between various antimicrobial agents, like nanoparticles and plant natural products, may provide new weapons to combat antibiotic resistant pathogens. The objective of this study was to examine the potential of silver nanoparticles (AgNPs) to enhance the antimicrobial activity of selected naphthoquinones (NQs): plumbagin (PL), ramentaceone (RAM), droserone (DR), and 3-chloroplumbagin (3ChPL). We also attempted to elucidate the mechanism by which the AgNPs enhance the antimicrobial activity of NQs. We analyzed the interaction of AgNPs with bacterial membrane and its effect on membrane stability (TEM analysis, staining with SYTO9 and propidium iodide), as well as aggregation of NQs on the surface of nanoparticles (UV-Vis spectroscopy and DLS analysis). Our results demonstrated clearly a synergistic activity of AgNPs and three out of four tested NQs (FBC indexes ≤ 0.375). This resulted in an increase in their combined bactericidal effect toward the *S. aureus* reference strain and the clinical isolates, which varied in resistance profiles. The synergistic effect (FBC index = 0.375) resulting from combining 3ChPL with silver nitrate used as a control, emphasized the role of the ionic form of silver released from nanoparticles in their bactericidal activity in combination with NQs. The role of membrane damage and AgNPs-NQ interactions in the observed synergy of silver nanoparticles and NQs was also confirmed. Moreover, the described approach, based on the synergistic interaction between the above mentioned agents enables a reduction of their effective doses, thus significantly reducing cytotoxic effect of NQs toward eukaryotic HaCaT cells. Therefore, the present study on the use of a combination of agents (AgNPs-NQs) suggests its potential use as a possible strategy to combat antibiotic-resistant *S. aureus*.

## Introduction

The effort placed on the exploration of new anti-infectious approaches is justified in the near post-antibiotic era that we face (Ventola, 2015). Drug resistance is observed in a growing number of clinical and environmental isolates of bacteria and fungi. Moreover, the problem of increasing antibiotic resistance concerns most of antibiotic drugs approved to treat infectious diseases (Magiorakos et al., 2012). *Staphylococcus aureus* is a Gram-positive bacterium belonging to a group of the most troublesome antibiotic resistant pathogens, so-called “ESCAPE" according to Peterson (2009). Most infections caused by *S. aureus* are associated with strains resistant to β-lactams (and also other classes of antibiotics) and most of them are healthcare-acquired like burn wound infections (Tong et al., 2015). The likelihood of anti-infectious treatment failing due to the antibiotic resistance is an increasingly recurring problem. In order to address those issues, there is an urgent need for the development of new, alternative approaches.

**FIGURE 1:**
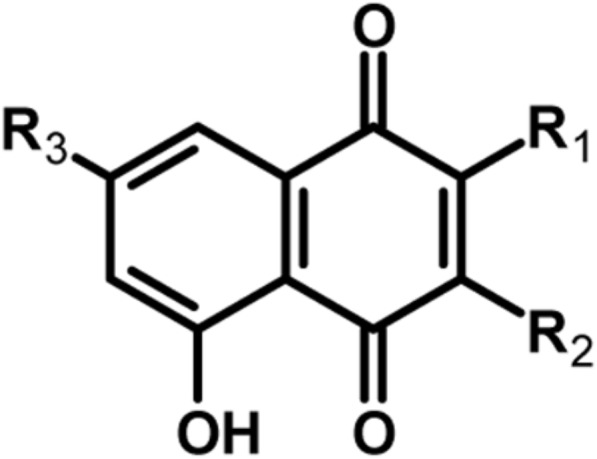
Chemical structure of four naphthoquinones selected for this study, present in tissues of carnivorous plants. Plumbagin: R_1_ = CH_3_, R_2_ = H, R_3_ = H; 3-chloroplumbagin: R_1_ = CH_3_, R_2_ = Cl, R_3_ = H; droserone: R_1_ = CH_3_, R_2_ = OH, R_3_ = H; ramentaceone: R_1_ = H, R_2_ = H, R_3_ = CH_3_.

Nanotechnology is a fast-developing field which can be applied to diverse medical issues. Silver nanoparticles (AgNPs), a typical example of nanomaterials, are widely studied because of their antimicrobial activity (Rai et al., 2009). Different formulations containing AgNPs have been proposed so far as antimicrobial treatments for burn wound infections (Jain et al., 2009), to improve wound healing (Tian et al., 2007), control implant infections (Juan et al., 2010), or to avoid medical device-related infections (Roe et al., 2008). The mechanism of antimicrobial action of AgNPs is complex and depends on both nanoparticles and silver ions released from their surface, and involves an interaction with many cellular components (Dakal et al., 2016). Many natural compounds of plant origin also possess antimicrobial activity and have a potential to fight antibiotic-resistant pathogens (Phoenix et al., 2014). In our previous study we demonstrated synergistic activity of an extract from *Drosera binata* when combined with AgNPs toward *S. aureus* (Krychowiak et al., 2014). Naphthoquinones (NQs), a group of secondary metabolites with a naphthalene backbone, were found to be the most prevalent and active constituents among all secondary metabolites detected in the extract studied. Droserone, 3-chloroplumbagin, plumbagin, and its isomer ramentaceone (**Figure 1**) are the most prevalent naphthoquinones synthesized in tissues of plants of the Droseraceae family (Juniper et al., 1989; Kreher et al., 1990; Gaascht et al., 2013; Krychowiak et al., 2014). The antibacterial activity of most of NQs concerns mainly Gram-positive bacteria like *S. aureus*, as most of Gram-negative bacteria are intrinsically resistant to NQs (Riffel et al., 2002; Krolicka et al., 2008, 2009; Moreira et al., 2017).

Although the direct use of naphthoquinones as antibacterial agents is limited due to their cytotoxicity toward the eukaryotic cells (Babich and Stern, 1993), their potential as alternative antimicrobials in an era of growing antibiotic resistance is worth exploring. Thus, we employed an approach based on a combination of antimicrobials to verify the possible use of NQs as anti-staphylococcal agents. Our study aims to examine AgNPs as agents that enhance bactericidal potential of NQs and in consequence reduce the effective dose of these secondary metabolites. Moreover, to investigate the possible mechanism of the observed synergistic effect, we also verified the hypothesis of the mode of synergistic action of AgNPs and NQs based on cell membrane damage caused by nanoparticles and their interaction with naphthoquinones.

## Materials and Methods

### Bacterial Strains and Antimicrobial Agents

In this study we used one reference strain of *S. aureus* (ATTC 25923) and four strains isolated from patients (Laboratory of Microbiology at the Provincial Hospital in Gdańsk, Poland), with different antibiotic resistance profile established according to CLSI guidelines (CLSI, 2012) for oxacillin, vancomycin and ciprofloxacin (Supplementary Table 1). The clinical isolates were described as follows: 703 k (oxacillin-resistant), 614 k (oxacillin- and ciprofloxacin-resistant), 56/AS (vancomycin- and ciprofloxacin-resistant), 6347 (oxacillin-, vancomycin-, and ciprofloxacin-resistant). All bacterial strains used in this study are stored in the Laboratory of Biologically Active Compounds, Department of Biotechnology, IFB UG and MUG Gdańsk, Poland.

Vancomycin, ciprofloxacin, oxacillin and plumbagin (PL) were purchased from Sigma-Aldrich and Daptomycin (DAP) from Selleck Chemicals. Protegrin-1 (PR) was synthesized by Lipopharm Sp. z o.o. (Poland). Ramentaceone (RAM) was obtained from the University of Pretoria, Republic of South Africa. Droserone (DR) and 3-chloroplumbagin (3ChPL) were synthesized at the Technical University of Gdańsk by E. Paluszkiewicz, Ph.D., as described by Krychowiak et al. (2014).

AgNPs solutions were purchased from Prochimia Surfaces Sp. z o.o. (Poland). As described by the manufacturer, AgNPs are water soluble, spherical nanostructures coated with (11-Mercaptoundecyl)-*N,N,N*-trimethylammonium chloride, characterized by the average size of 5.5 nm and dispersity level of 15%. The initial concentration of AgNPs solutions was 6.74 × 10^14^ NPs/ml equivalent to 615 μg/ml (SPR maximum, aaa_max_: 420–424 nm).

### Antibacterial Assays

To characterize antibacterial potential of all agents tested in this study, alone and in combinations, we determined their minimal bactericidal concentration (MBC), which is the lowest concentration of agent that after 24 h reduces the number of bacterial cells in the initial inoculum by 99.9% (3 logarithms). To determine the MBC we used the Microdilutions Broth Method (Thornsberry, 1991) according to the CLSI guidelines (CLSI, 1996). To that effect, we prepared twofold dilutions of the tested agents in 96-well plates, with final volume of 0.1 mL per well. The tested concentrations ranged from 0.125 to 512 μg/mL. Bacterial inoculum was prepared from late-log cultures (37°C, 150 rpm) in Cation-adjusted Mueller-Hinton Broth (CA-MHB) prepared from colonies on BHI agar plates (24 h, 37°C). Liquid culture was diluted in fresh CA-MHB medium to 0.5 McF (according to McFarland standards) measured with a densitometer (DensiMeter II, EMO, Brno), corresponding to ∼1.5 × 10^8^ CFU/mL (colony forming units per mL). The bacterial suspension was then diluted with medium to a density of 2.5–5 × 10^6^ CFU and 10 μL aliquots were allocated to each well of the 96-well plates. In addition, dilutions containing untreated bacteria were plated out to check the number of bacterial cells in wells in each experiment. The 96-well plates were then incubated without shaking for 24 h at 37°C. Wells with no visible growth of bacteria were plated out on BHI agar plates and incubated for 24 h at 37°C, colony counts made to determine the lowest bactericidal concentration of each agent, or combination of agents. Each concentration or combination of concentrations was tested in triplicate and each experiment was performed at least in triplicate.

### Synergy Testing

In order to examine the interaction mechanism of tested combinations of antimicrobials (synergistic, additive or antagonistic) we employed the Checkerboard Titration Method (Thornsberry, 1991). This method is based on testing the combination of two agents at their concentration gradient obtained by twofold dilutions. The range of the tested concentrations of each agent was from 0.03× MBC to 2× MBC. Such an approach allowed determination of changes (reduction or increase) in the minimal effective concentrations of the analyzed agents after their combination. The mode of action of two agents used simultaneously is expressed mathematically as the Fractional Bactericidal Concentration index (FBC index), calculated according to the equation: FBC index = FBC_A_/MBC_A_ + FBC_B_/MBC_B_, where MBC_A_ and MBC_B_ are the lowest bactericidal concentrations of A and B tested separately, whereas FBC_A_ and FBC_B_ are the lowest bactericidal concentrations of agent A and B when they are used in combination. The nature of interaction of two agents is described by the value of FBC index: lower or equal to 0.5 – synergistic interaction, higher than 0.5 but lower or equal to 1 – additive interaction, higher than 1 – antagonistic interaction. The isobole method (Barenbaum, 1978) was used to visualize the character of interaction between AgNPs and NQs whereby the isobole curve being a line connecting the points of the lowest bactericidal concentrations of the combined agents.

### Time-Dependent Killing

In each step of time-dependent killing assay, the bacterial cells were cultured aerobically in CA-MHB medium at 37°C and 150 rpm. Bacterial cells grown overnight were diluted 100 times in fresh medium and cultured for another 4 h (37°C, 150 rpm) until they reached a mid-log phase. Then, bacterial suspension was diluted in a fresh medium to 0.5 McF measured with a densitometer (DensiMeter II, EMO, Brno), 0.1 mL aliquots of inoculum were then transferred to wells on 24-well plate with 1 mL of medium containing the tested antimicrobials (8 μg/mL 3ChPL or 3.6 μg/mL AgNPs) and their combinations (8 μg/mL 3ChPL combined with 3.6 μg/mL AgNPs). Wells containing medium without antimicrobials were treated as control. Each well was sampled (50 μL) for colony counts at the following timepoints: 0, 1, 2, 4, 6, and 24 h and the plates were incubated as described above. Each concentration of agent, or combination of agents, was tested in triplicate and each experiment was performed at least in triplicate.

### Membrane Damage

The level of membrane integrity was studied by staining with SYTO9 and propidium iodide (PI) according to method described by O’Neill et al. (2004) with modifications. The principle of this method is to use two probes staining nucleic acids: a cell permeable dye (SYTO9) and an impermeant probe (PI). The permeability of PI is enhanced when cells are dead or their membrane is impaired. Overnight culture of bacterial cells in CA-MHB medium was diluted 100 times in fresh medium and cultured for next 6 h at 37°C with agitation (150 rpm). Bacterial cells were centrifuged (2,800 ×*g*, 10 min), washed twice and finally diluted in physiological saline (PS) to 0.5 McF (DensiMeter II, EMO, Brno). The inoculum samples were then mixed with the tested agents (AgNPs, DAP, PR) to final concentration of 0.5× MBC. Both DAP and PR, two membrane disrupting agents, were used separately as positive controls. Bacterial suspension without any antimicrobial agent added was taken as an untreated control. Inoculum aliquots (0.1 mL) were transferred immediately into the wells of flat-bottom black polystyrene 96-well plate (FluoroNunc^TM^, Thermo Fisher Scientific). Following incubation at 37°C (30, 60, and 120 min, without shaking, cultures in wells were combined with 0.1 mL aliquots of mixed solution of dyes in PS: 60 μM of PI (Sigma Aldrich) and 10 μM of SYTO9 (Thermo Fisher Scientific). After stationary incubation (in the dark, RT, 15 min) fluorescence of green dye (SYTO9) and red dye (PI) was measured on a microplate reader (EnVision Multilabel Plate Reader, Perkin Elmer) at Ex/Em = 485/530 and Ex/Em = 485/630, respectively. Ratio of green to red fluorescence was calculated for all samples. The values obtained for samples treated with agents were compared to those obtained for untreated control (corresponding to 100% membrane stability in each time point). To control the number of living bacteria we performed colony counts for each sample. Each agent was tested in three replicates and whole experiment was performed at least in triplicate.

### Analysis of Structure of Bacterial Cells With Transmission Electron Microscopy

Transmission electron microscopy (TEM) was used to determine changes in structure of bacterial cells treated with AgNPs (or AgNO_3_), 3ChPL, and their combination. Inocula in late log phase (cultured for 6 h, at 37°C and 150 rpm) in CA-MHB medium (0.5 McF, 1.5 × 10^8^ CFU/mL) were treated with agents, or combinations of agents, at concentration corresponding to 5× MBC for 90 and 180 min (37°C, 150 rpm). Following the treatment, the bacterial cells were centrifuged (2,800 ×*g*, 10 min) and washed twice with phosphate-buffered saline. Bacterial pellets left in tubes were fixed with 2.5% glutaraldehyde (Polysciences, Warrington, PA, United States), and then with 1% osmium tetroxide (Polysciences, Warrington, PA, United States). After ethanol dehydration, bacteria were embedded in Epon 812 resin (Sigma-Aldrich). The ultramicrotome Leica UC7 was used to prepare ultrathin sections (55 nm). Lead citrate and uranyl acetate were added as contrasting agents. The entire study was performed at 120 kV using Tecnai Spirit BioTWIN microscope (FEI).

### Analyses of Interactions of NQs and AgNPs

To analyze the interactions between AgNPs and a selected naphthoquinone (3ChPL), we measured light absorption spectra in a wide wavelength range of 300–800 nm with 0.5 nm intervals, using SPECORD 50 Plus Analytik Jena spectrophotometer with a thermostat (25 ± 0.1°C). Three series of spectrophotometric titrations were performed: (i) buffer titrated with an increasing amount of tested compounds (concentration range from 1 to 20 μg/mL for 3ChPL and from 1.8 to 7.4 μg/mL for AgNPs), (ii) buffer containing 3ChPL (initial concentration 20 μg/mL) titrated with AgNPs (concentration range from 1.8 to 7.4 μg/mL), and (iii) buffer containing tested NQ (initial concentration 20 μg/mL) titrated with distilled water in a volume corresponding to the volume of AgNPs solution. Experiments were done in quartz cuvettes (1 cm light path) containing 2 mL of 0.2 M sodium-phosphate buffer pH 6.8.

The analyses of the size distribution of aggregates in ddH_2_O for AgNPs alone (12.8 μg/mL) and AgNPs with 3ChPL (12.8 μg/mL with 8 μg/mL, respectively) were performed by dynamic light scattering (DLS) measurement on Zetasizer Nano ZS (Malvern, Worcestershire, United Kingdom), by measuring the intensity of the scattered light. The analyzed solutions were placed in polystyrene cuvettes. Measurements were conducted at 25°C with a He–Ne laser (633 nm, 4 mW), at a 173° scattering angle. Results were evaluated using Smoluchowski approximation, which is known to be rigorously valid only for spherical-like particles. The obtained data are shown as size distribution [nm] of light scattering particles (in accordance to their hydrodynamic diameters) by intensity [%].

### Evaluation of Cytotoxicity on Eukaryotic Cell Line

The human skin keratinocyte cell line HaCaT (CLS order no. 300493) was used to assess the cytotoxicity of tested agents, and their combinations, by employing the MTT assay, according to the protocol of Krychowiak et al. (2014). Cell cultures were treated with a concentration gradient ranging from 1× MBC to 0.03× MBC of 3ChPL, AgNPs, or AgNO_3_, as well as with concentration gradient of 3ChPL combined with AgNPs (0.25× MBC). The absorbance of formazan was measured at 550 nm using a microplate reader (Victor 2, 1420 Multilabel Counter, Perkin Elmer). The cells survival rate was calculated according to the equation: survival rate (%) = (A – A_B_/A_C_ – A_B_) × 100, where A was the absorbance value of treated sample, A_B_ was the absorbance value of blank sample (untreated cells, without formazan salt) and A_C_ was the absorbance of untreated sample.

### Statistical Analysis

The results of biological assays were analyzed for statistical significance with the Statistica 13 software (StatSoft). The one-way analysis of variance (ANOVA) followed by the *post hoc* RIR Tukey’s test was applied. For pairwise comparisons, a paired Student’s *t*-test was performed. Significance level was established at α = 0.01.

## Results

### Evaluation of Synergistic Activity of AgNPs and NQs

**FIGURE 2:**
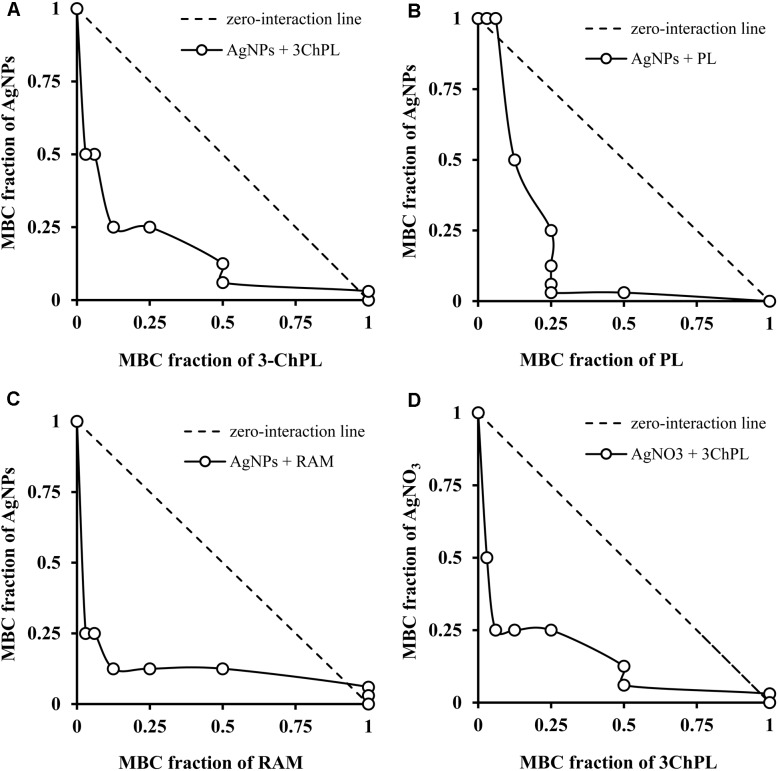
Isobole curves depicting synergistic bactericidal effect of silver nanoparticles (AgNPs) and selected naphthoquinones toward *Staphylococcus aureus* strain ATCC 25923. **(A)** Combinatorial effect of 3-chloroplumbagin (3ChPL) and AgNPs; **(B)** combinatorial effect of plumbagin (PL) and AgNPs; **(C)** combinatorial effect of ramentaceone (RAM) and AgNPs; **(D)** combinatorial effect of 3ChPL and silver nitrate (AgNO_3_).

The checkerboard Titration Method employed to test the bactericidal potential of combined NQs and AgNPs revealed their synergistic interaction in the isobole curves (**Figures 2A–C**). Only droserone (MBC = 512 μg/mL) was unable to interact in a synergistic manner with the AgNPs (FBC index = 1.03; data not shown). RAM, PL and 3ChPL, all with high anti-staphylococcal activity (MBC equal to 16, 16, and 8 μg/mL, respectively), exhibited synergistic bactericidal effect when combined with AgNPs. For these three NQs the isoboles were placed under the zero-interaction line and were concave in shape. Moreover, the FBC indices for all of the compounds were lower than 0.5 (0.28 for PL and RAM, 0.375 for 3ChPL). Noteworthy is the fact that when the bacterial cells were treated with combined AgNPs and NQs, the bactericidal effect was observed at significantly lower concentrations for all of the tested agents. The effective doses of naphthoquinones were reduced by 75–97%. At the same time, bactericidal concentration of AgNPs used simultaneously with NQs decreased by 75–97%. In further experiments we chose the most active 3-ChPL (MBC = 8 μg/mL) as a representative and tested the combination of AgNO_3_ and 3ChPL on *S. aureus* cells. According to the results shown in **Figure 2D**, silver ions also interact with 3ChPL (concave curve, under the zero-interaction line) and the FBC index (0.31) was slightly lower than FBC index for AgNPs combined with 3ChPL (0.375).

### Time-Dependent Killing Efficiency of Combination AgNPs-NQs

**FIGURE 3:**
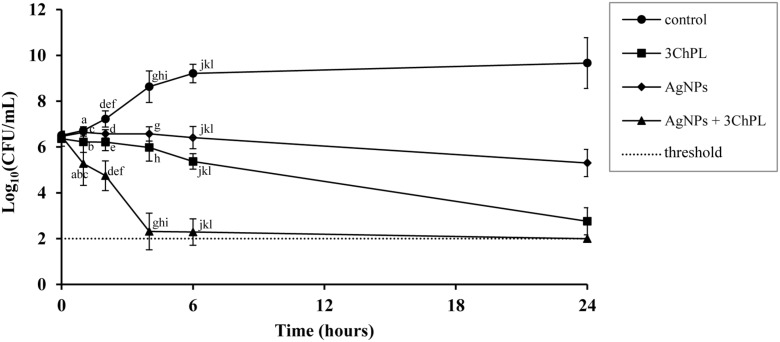
Changes in time-dependent killing efficiency of naphthoquinone after addition of AgNPs with the example of 3ChPL tested toward *S. aureus* strain ATCC 25923. Curves were obtained for concentrations of agents used alone or in combinations equal to 1× MBC (8 μg/mL) for 3ChPL and 0.25× MBC (3.6 μg/mL) for AgNPs. Results are reported as mean values of 9 replicates ± SD. Values indicated with similar letters are significantly different from each other (*p* < α, α = 0.01).

Experiments on time-dependent killing of bacterial cells confirmed the synergistic interaction of AgNPs and NQs (**Figure 3**). First of all, the number of bacterial cells treated with AgNPs at concentration corresponding to 0.25× MBC (3.6 μg/mL) combined with 3ChPL at its bactericidal concentration (8 μg/mL) dropped significantly. Furthermore, the inoculum size in wells supplemented with combination of agents was about four logarithms lower after just 4 h in comparison to samples treated with each agent separately.

### Anti-Staphylococcal Potential of AgNPs-NQs Combination Toward Antibiotic-Resistant Clinical Isolates

To examine the effect of interaction of AgNPs and 3ChPL on clinical isolates of *S. aureus* with different profiles of antibiotic resistance, experiments were carried out on bacterial cells treated with the given combination of agents. The results clearly depict that the synergistic effect is observed also for strains with resistance to antibiotics (**Table 1**). For all of the selected isolates the FBC index was equal to or even lower than 0.375 in spite of their resistance profile. This result emphasize the potential of the AgNPs-NQ combination to combat antibiotic-resistant strains of *S. aureus*.

**Table 1:** Summary of checkerboard assays for combination of 3-ChPL and AgNPs tested toward the reference strain of *S. aureus* and clinical isolates resistant to antibiotics.

*S. aureus* strains	FBC index	3ChPL		AgNPs	
		FBC (μg/mL)	Reduction of MBC (%)	FBC (μg/mL)	Reduction of MBC (%)
ATCC 25923	0.375	1	87.5	3.6	75
703 k^o^	0.375	0.5	87.5	0.9	75
614 k ^o,c^	0.375	2	87.5	0.45	75
56/AS ^c,v^	0.25	1	87.5	0.45	87.5
6,347^o,c,v^	0.375	0.5	87.5	0.9	75

o, oxacillin-resistant; c, ciprofloxacin-resistant; v, vancomycin-resistant; FBC, fractional bactericidal concentration, bactericidal concentration in combination; MBC, minimal bactericidal concentration.

### *In Vitro* Cytotoxicity Assessment

**FIGURE 4:**
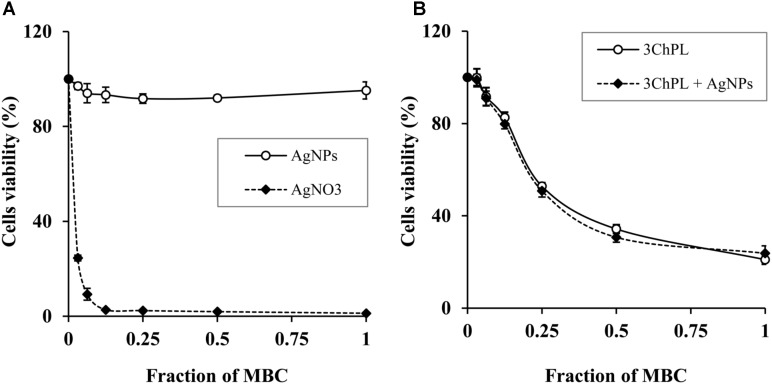
Dose-dependent changes of cytotoxicity of the tested agents toward HaCaT cells cultured *in vitro*. **(A)** Viability of cells treated with AgNPs and AgNO_3_ at range of their concentrations from 0.03× MBC to 1× MBC; **(B)** viability of cells treated with 3ChPL in range of its concentration (0.03×MBC to 1× MBC) both alone and combined with AgNPs at 0.25× MBC. Minimal bactericidal concentrations (1× MBC) of agents tested separately were equal to 14.4 μg/mL (AgNPs), 8 μg/mL (3ChPL), and 16 μg/mL (AgNO_3_).

The aim of these experiments was to assess the preliminary therapeutic potential of AgNPs-NQs. The results obtained for eukaryotic cells treated with different forms of silver clearly demonstrated that silver nanoparticles were non-toxic toward eukaryotic cells in the tested range of concentrations (from 0.03× MBC to 1× MBC) whereas silver nitrate was highly cytotoxic toward the HaCaT cells (**Figure 4A**). Cell viability was significantly decreased (24.54 ± 1.20%) even when they were challenged with the lowest tested concentration of AgNO_3_ (0.5 μg/mL). It is also worth emphasizing that IC_50_ value of AgNO_3_ was extremely low (<0.5 μg/mL) and significantly lower than the IC_50_ value of 3ChPL (2.2 μg/mL) (**Figure 4B**).

The viability measured by the MTT assay was relatively low when the cells were treated with 3ChPL at its minimal bactericidal concentration (MBC = 8 μg/mL) as shown in **Figure 4B**. However, the survival rate of HaCaT cells was significantly higher when concentrations of 3ChPL ranged from 0.25 to 1 μg/mL. A subsequent experiment carried on HaCaT cells treated with the same concentration range of 3ChPL but supplemented with AgNPs (0.5× MBC = 7.2 μg/mL), revealed that the toxicity of the NQ was not enhanced by AgNPs.

### Damage Detection in Bacterial Cell Membrane

**FIGURE 5:**
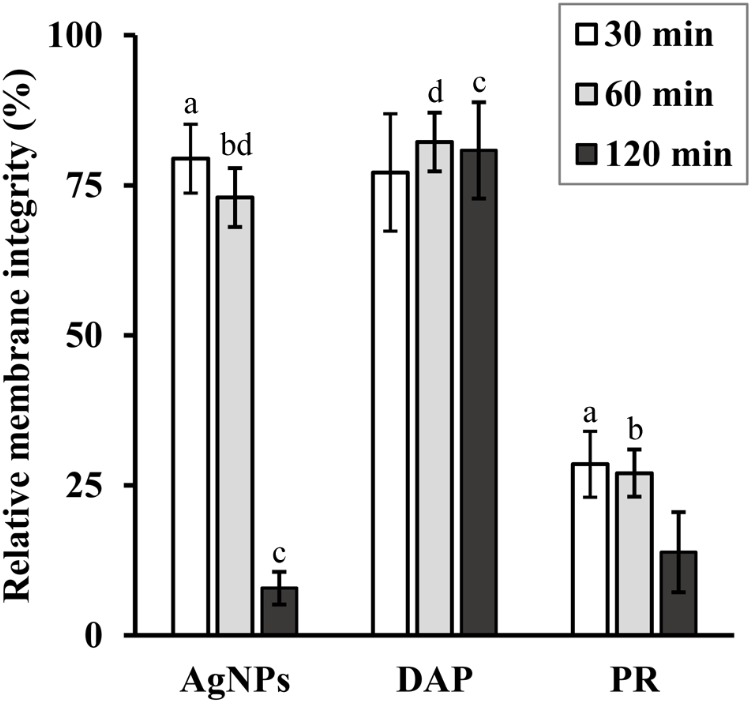
Time-dependent damage of bacterial cell membrane after treatment with AgNPs, daptomycin (DAP) and protegrin (PR) at their concentrations corresponding to 0.5× MBC. Experiments were performed on *S. aureus* strain ATCC 25923. Results are reported as mean values of 9 replicates ± SD. Values indicated with similar letters are significantly different from each other (*p* < α, α = 0.01).

The procedure of cell staining with SYTO9 and PI was used to determine the bacterial cell membrane stability after treatment with AgNPs. Two antimicrobials, DAP and PR, known for their potential to interact and disrupt bacterial cells membranes, were used as a control. Each agent was tested at a concentration equal to 0.5× MBC (AgNPs: 6.2 μg/mL, DAP: 4 μg/mL, PR: 8 μg/mL) to follow the changes during time course experimentation (**Figure 5**). AgNPs led to a slight drop in membrane integrity during the first 60 min of incubation (membrane integrity level equal to 72.95 ± 4.91%), followed by a statistically significant disruption after 120 min (membrane integrity level equal to 7.85 ± 2.72%). The curve representing the results obtained for DAP used as a control agent, showed only a slight drop over the entire time course (to 80.81 ± 8.02% after 120 min). The integrity of bacterial cell membranes treated with PR was significantly impaired after 30 min and slightly decreased after next 90 min (to 13.84 ± 6.67%). To confirm that decreased ratio of green to red fluorescence did not correlate with bacterial cells killing, we prepared cell counts after 120 min treatment. The average number of bacterial cells after 120 min incubation without and with AgNPs, DAP and PR was 6.87 × 10^6^, 4.8 × 10^6^, 6.23 × 10^6^, 5.33 × 10^6^ CFU/mL, respectively.

**FIGURE 6:**
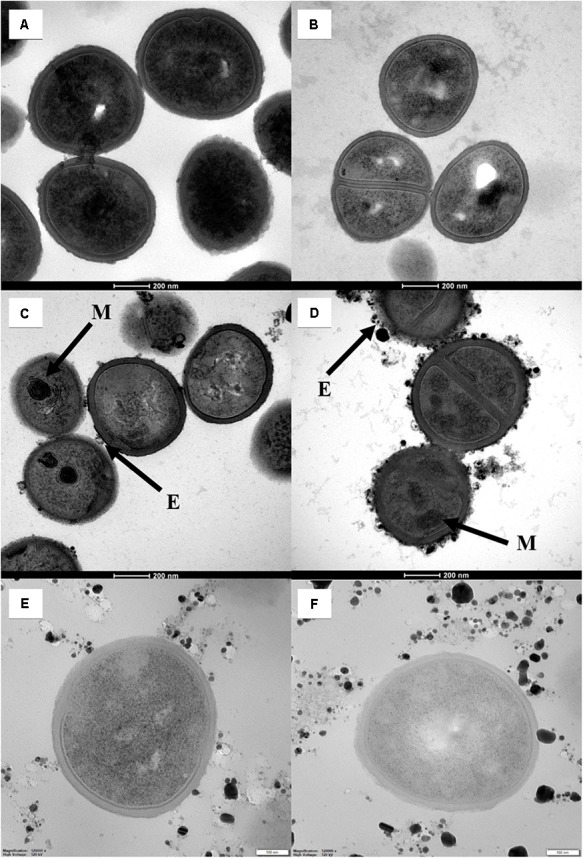
Structure of bacterial cells treated with particular agents or their combinations for 180 min. **(A)** Untreated cells; **(B)** cells treated with 3ChPL at 5× MBC concentration (40 μg/mL); **(C)** cells treated with AgNPs at 5× MBC concentration (72 μg/mL); **(D)** cells treated with combination of 3ChPL and AgNPs (each at concentration 5× MBC); **(E)** cells treated with AgNO_3_ at 5× MBC concentration (80 μg/mL); **(F)** cells treated with combination of 3ChPL and AgNO_3_ (each at concentration 5× MBC); Experiments were performed for the *S. aureus* strain ATCC 25923. Black arrows depict structural changes in bacterial cells: M, mesosome-like structures; E, exosome-like structures.

Subsequent TEM analysis of bacterial cell structure confirmed the aforementioned changes in membrane integrity (**Figures 6A,C,D**). The surface of *S. aureus* cells treated with the AgNPs (alone or in combination with 3ChPL) was covered with aggregates of nanoparticles and the AgNPs clusters were also found inside the exosome-like structures made of cell membrane fragments. Moreover, the way of folding of the intracellular membrane was observed and most of the bacterial cells contained mesosome-like structures. Only small changes (moderate aggregation in the bacterial cytoplasm) appeared in bacterial cells treated with 3ChPL alone (**Figure 6B**). When 3ChPL was used simultaneously with AgNPs, the structural changes were similar to those observed for cells treated with AgNPs alone. Alterations in the structure of bacterial cells treated with AgNO_3_ (alone and in combination with 3ChPL) were subtle (**Figures 6E,F**). We observed only weak internal aggregation in the cells. Nanoparticles presented in **Figures 6E,F** were formed from AgNO_3_ reduced in the applied culture conditions.

### Analysis of Interactions of AgNPs and NQ

**FIGURE 7:**
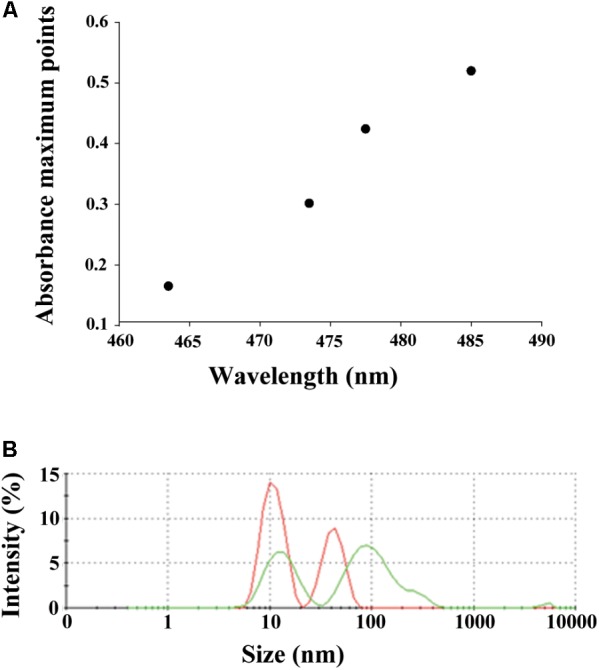
Results of analyses of the interactions of AgNPs and naphthoquinones. **(A)** The changes of the maximum absorbance points of spectra of the 3ChPL (initial concentration 20 μg/mL) titrated with AgNPs (concentration range 1.8–7.4 μg/mL). **(B)** Changes in hydrodynamic diameter size of nanoparticles measured by DLS in the presence of 3ChPL. Red line corresponds to average hydrodynamic diameter size distribution in a polydispersive population of AgNPs (initial concentration 12.3 μg/mL), peaks size: 10.96 and 42.63 nm. Green line represents the average hydrodynamic diameter size distribution of mixture containing AgNPs and 3ChPL (final concentrations 12.2 and 8 μg/mL, respectively), peaks size: 13.61 and 119.9 nm.

To verify whether the AgNPs and 3ChPL interact (directly and non-covalently), we analyzed their absorbance spectra alone and in combination. Since the overlapping spectra of NQ and AgNPs prevent complete thermodynamic analysis, spectra registered for 3ChPL were subtracted from those registered for 3ChPL titrated with silver nanoparticles (concentration range: 1.8–9.2 μg/mL). Significant red shifts in the maxima of absorbance obtained for AgNPs added to buffer containing 3ChPL, suggest that these two agents do interact (**Figure 7A**). Additionally, the analysis of changes in the hydrodynamic diameter of AgNPs in the presence of NQ confirmed this hypothesis (**Figure 7B**). The average hydrodynamic diameter of nanoparticles increased from 10.96 and 42.63 nm to 13.61 and 119.9 nm, respectively, when 8 μg/mL of 3ChPL was added to an aqueous solution of AgNPs.

## Discussion

Since the emergence of drug resistance, a continuous development of new therapeutic approaches for microbial infections has become essential. Strategies based on synergistic combinations of antimicrobial agents, as well as drug design based on nanotechnology and phytomedicine can be effective in overcoming microbial resistance. The scope of our study was to explore the synergistic potential of the selected NQs of plant origin and AgNPs toward *S. aureus.* As little is known about the mechanism underlying the synergistic effect of AgNPs-NQs combination, we also verified the role of the main mechanistic aspects of the bactericidal activity of AgNPs in the observed synergy when the nanoparticles are used simultaneously with naphthoquinone.

In this study we determined the *in vitro* synergistic anti-staphylococcal activity of the AgNPs and selected NQs, as well as verified the potential of this combination toward the clinical isolates of *S. aureus* with distinct antibiotic resistance profile. The bactericidal potential of three out of the four studied NQs was significantly enhanced by AgNPs. 3-chloroplumbagin, as the most potent anti-staphylococcal naphthoquinone relative to PL and RAM, was selected for further testing. A synergistic bactericidal effect was not observed when AgNPs were combined with DR. Compared to PL, RAM, and 3ChPL, the chemical structure of DR is characterized by an additional hydroxyl group. It has already been demonstrated that a number of polar groups, like OH- residue, play an important role on both polarity and biological activity of NQs (Munday et al., 2007; Camara et al., 2008; Castro et al., 2008). The relationship between the structure of NQs and the synergistic interaction with the AgNPs along with *in vivo* studies on toxicity and anti-infectious potential will be further investigated. The synergistic bactericidal effect observed for 3ChPL and silver nitrate indicates that silver ions can play an important role in the enhancement of NQs’ bactericidal activity. It confirms that the direct toxic effect of AgNPs on microbial cells depends on silver ions appearing inside the cell and in the cell milieu where metallic silver is oxidized to silver ions (Xiu et al., 2012). Time-dependent killing efficiency of 3ChPL was significantly enhanced when 3ChPL was combined with AgNPs, thus confirming synergistic potential of the AgNPs-NQ combination. Moreover, the combination of AgNPs and 3ChPL examined was effective on the clinical isolates with different resistance profiles. All clinical isolates, resistant to one or more antibiotics, were susceptible to the combined treatment.

Moreover, we employed the MTT assay to measure the cytotoxic effect of the tested antimicrobials and their combination toward eukaryotic cell cultures. The aim of the analysis was to preliminarily evaluate the potential role of AgNPs-NQ as antimicrobial agents. The differences in toxicity of AgNPs and AgNO_3_ confirmed that silver nanostructures can be considered as being non-toxic, sources of anti-infectious Ag^+^ ions that are released from their relatively large surface (Raffi et al., 2008). What is more, 3ChPL occurred to be less toxic at and below the value of its fractional bactericidal concentration in combination with AgNPs. Furthermore, the addition of AgNPs to the samples treated with 3ChPL did not affect the viability of cells. These results highlight the potential of the nanoparticles of silver: not only they improve the antimicrobial activity of NQ but also help to fine-tune its dose.

To assess the mechanism of the observed synergy phenomenon, we investigated the role of the disruption of bacterial cell membrane by AgNPs. The experiments on cells stained with SYTO9/PI revealed that AgNPs significantly disrupt *S. aureus* cell membrane with a rate similar to protegrin-1, a peptide used as a one of the membrane-disrupting agents in positive controls. What is more, protegrin-1 combined with 3ChPL gave an additive bactericidal effect and reduced bactericidal concentration of this naphthoquinone by 75% (FBC index = 0.75, data not shown). On the contrary, daptomycin did not disrupt the membrane significantly and did not interact with 3ChPL (FBC index = 1.03, data not shown). This suggests that cell membrane disruption might be one of the mechanisms that underlie the synergistic effect of AgNPs and NQs. Since the role of the membrane is to protect bacterial cell from penetration and adverse effects of toxic molecules, decrease of membrane integrity would allow enhanced permeation and likely cell death. For hydrophobic molecules like naphthoquinones, such mechanism plays an undeniable role in the enhancement of the antimicrobial potential (Nikaido, 2003). TEM micrographs obtained in our study confirmed the changes in bacterial cell membrane after treatment with AgNPs. Cells treated with nanoparticles were covered with silver aggregates, formed external vesicles containing metallic silver and internal mesosome-like structures, also, they had visible agglomerates in the cytoplasm. Although bacterial mesosomes are considered to be artificial structures formed from cell membrane during sample preparation procedure (Silva et al., 1976), their occurrence had been reported in bacterial cells treated with agents that impair cytoplasmic membrane (Shimoda et al., 1995; de León et al., 2010; Rabanal et al., 2015).

We employed a simple method based on UV-Vis spectroscopy, followed by DLS measurement to verify whether the AgNPs interact directly with NQs. Shifts in the absorbance spectra of NQs titrated with AgNPs along with the extension of hydrodynamic diameter of two populations of nanoparticles homoaggregates in the presence of 3ChPL demonstrate interactions between the studied agents. AgNPs used in this study were spherical nanostructures stabilized with thioalkane chains which are responsible for the interaction of nanoparticles with other chemical compounds (Rana et al., 2012). Furthermore, it is widely known that the physical interaction (formation of complexes) of drug and nanoparticles modulates activity and can play an important role in the final therapeutic effect (Deng et al., 2016).

Synergistic strategies have been widely studied and successfully used for many approved pharmaceuticals. In this paper, we present an approach based on the synergistic activity of AgNPs and naturally occurring compounds. To the best of our knowledge, this is the first report exploring enhanced antimicrobial potential of NQs-AgNPs combination, and also describes its possible mode of action. The synergistic activity in this combination has resulted in significant enhancement of bactericidal activity regardless of antibiotic resistance, together with a reduction of cytotoxic effect toward the eukaryotic cells, as well as in a final multi-target antimicrobial effect. Through the release of silver ions, interacting with the molecules of naphthoquinone and disruption of bacterial cell membrane, the AgNPs enhance the NQs’ anti-staphylococcal activity. Such complex mechanism of bacterial cells killing reduces the probability of resistance development (Ulrich-Merzenich et al., 2009). Synergy testing is an extremely valuable research field that allows for the design of drug composition active toward antibiotic-resistant pathogens like *S. aureus*.

## Conclusion

In this study, we have demonstrated the potential of AgNPs to enhance the bactericidal activity of three NQs toward *S. aureus*. Moreover, our approach was also effective with bacterial isolates resistant to many antibiotics. It is a first report which includes the analysis of the mechanism responsible for the evaluation of the anti-staphylococcal activity of NQs. The complexity and efficiency of the combinations examined could be of value in era of bacterial multi-drug resistance. The combination of nanotechnology and phytopharmacy is fast becoming a new research field that is worth exploring and expanding in order to design new possible treatments for infectious diseases.

## Data Availability

All data supporting the conclusions of this manuscript will be made available on request.

## Author Contributions

MK and AKr conceived the original idea, designed the study, and wrote the manuscript. MK, AKa, MN, and AB performed the experiments. MK analyzed the data. AB performed the statistical analysis.

## Conflict of Interest Statement

The authors declare that the research was conducted in the absence of any commercial or financial relationships that could be construed as a potential conflict of interest.
