# Supplementary material for: Silver Nanoparticles Combined With Naphthoquinones as an Effective Synergistic Strategy Against Staphylococcus aureus
Source: Front Pharmacol. 2018 Jul 26;9:816. doi: 10.3389/fphar.2018.00816 (PMC6094968; doi:10.3389/fphar.2018.00816)
Supplement: Supplementary file 1 [file Table_1.DOCX]

**Supplementary Table 1.** Antimicrobial potential of all tested antibiotics and agents.

| *S. aureus* strain | MIC (µg/mL) | | | MBC (µg/mL) | | | | | | | |
| --- | --- | --- | --- | --- | --- | --- | --- | --- | --- | --- | --- |
|  | OXA | VAN | CIP | AgNPs | AgNO_3_ | 3ChPL | PL | RAM | DR | DAP | PR |
| ATCC 25923 | N/A | N/A | N/A | 14.4 | 16 | 8 | 16 | 16 | 512 | 8 | 16 |
| 703k | 64 | 2 | 1 | 3.6 | N/A | 4 | N/A | N/A | N/A | N/A | N/A |
| 614k | >128 | 2 | >128 | 18 | N/A | 16 | N/A | N/A | N/A | N/A | N/A |
| 56/AS | 1 | 4 | 64 | 3.6 | N/A | 8 | N/A | N/A | N/A | N/A | N/A |
| 6347 | >128 | 4 | 16 | 3.6 | N/A | 4 | N/A | N/A | N/A | N/A | N/A |

OXA – oxacillin, VAN – vancomycin, CIP – ciprofloxacin, AgNPs – silver nanoparticles, AgNO_3_ – silver nitrate,

3ChPL – 3-chloroplumbagin, PL – plumbagin, RAM – ramentaceone, DR – droserone, DAP – daptomycin, PR – protegrin-1; N/A – not applicable.
